# Complexity of mentalization

**DOI:** 10.3389/fpsyg.2024.1353804

**Published:** 2024-02-29

**Authors:** Zlatoslav Arabadzhiev, Rositsa Paunova

**Affiliations:** Department of Psychiatry and Medical Psychology, Medical University of Plovdiv, Plovdiv, Bulgaria

**Keywords:** mentalization, psychotherapy, mental disorders, stigmatization, etiology

## Abstract

The ability to understand other people’s emotions, thoughts, and behaviors is associated with an individual’s ability and his attitude in the context of relationships. The development of this process is associated with different psychosocial factors: parental competence, building a secure attachment and reduced risk of violence in the family as well as the future intrapersonal relationships and social reality of one person Mentalization is empirically associated with resolving psychological tasks in normative dilemmas and also, can influence on the outcome of personal and situational crises. And so forth, passing through the different stages of life and prevention of dysfunctional patterns in the family is facilitated. This text aims to investigate the main characteristics of mentalization, its connection with the etiopathogenesis of some main mental disorders, their course and prognosis. Several studies in recent years confirmed that these connections can enroll mentalization to prevention and psychological therapy in some mental disorders and dysfunctional thinking patterns. On the other hand, it serves as a basis for psychotherapeutic competence expressed in the ability to connect, differentiate, and separate, as the main interactional stages of the psychotherapeutic process.

## Introduction

The “psychological reading” of mentalization is directly related to the complexity and understanding as part of the personality (Fonagy and Allison, 2012). The meaning of early psychological experience unfolds in experiencing the actual situation and developing a personal repertoire of thinking, feeling, and behaving in the individual’s intra- and interpersonal world. The series of actual situations in the individual’s life only stabilize and expand the repertoire hidden in his capacity for mentalization. Based on this theoretical formulation, we can see the dynamics between biological, psychological, social and cultural factors influencing this process. It would also help to understand the influence of mentalization on predisposition, pathomorphosis, compliance, adherence to therapy and possibly the outcome of suffering. Mentalization is a mental activity process in which individuals perceive and understand their own as well as other people’s thoughts, feelings, and intentions. Moreover, it is associated with the individual stages of the volitional process (choosing a goal, fighting motives and counter-motives, implementing decisions). It is a term used to describe one person’s ability to see himself and others as individuals with an inner psychic life. This mental phenomenon includes a wide range of mental states, dimensions, and abilities (Fonagy and Luyten, 2009):

- Understanding the causes of one’s emotions, thoughts and behavior. This dimension is related to the focus and purpose of psychotherapy meetings (Varcheva, mentalization process when working with families, 2020), in order to bring up the individual’s consciousness to the relation between the current situation (or a re-enactment of a previous painful experience) and how he thinks, feels, and reacts. From this perspective, the individual can move on and explore the various options for alternative behaviors and thoughts, along with the reasons for the rigidity and inflexibility in the context of the patient’s/client’s life concepts and beliefs. Mentalization is related to the sense of identity, the ability to accept oneself, and “comfort in one’s skin.”
- Interpreting the thoughts, feelings and behavior of others – “putting yourself in another person’s shoes.” Having the ability to distance yourself from someone’s own experiences about the situation, to shift the focus away from someone’s own social and psychological experience, and also to have the ability to see the other person’s “whole picture.” In this line of thought, the high level of mentalization is associated with overcoming prejudice and accepting others in their wholeness and individuality without an overwhelming need for change.
- Understanding the complexity of mental life allows the existence of mixed and opposite feelings, including the deployment of abilities such as adaptability, flexibility, and predictability in various situations.
- Luyten and Fonagy (2015) divide the process of mentalizing into two types: automatic/implicit mentalizing, which is an energy-saving process, and controlled/explicit mentalizing, which requires mental energy because uses of active attention and thinking are involved (Fonagy et al., 2016). On the other hand, mentalizing can be based on actual observed behavior and circumstances or can be inferred from someone’s own experience of what is happening either hypothesis about the thoughts, emotions and intentions of the others (Fonagy and Target, 1996). Finding a balance between these two types of mentalizing can be a good potential to build up an ability in individuals by learning to seek feedback in terms of the external cues of the situation and their transference to it.

Mentalization is associated with various aspects of human behavior that arise spontaneously. It is a continuous process and not a personality characteristic. Mentalizing relates to empathy, building interpersonal relationships, and emotional and social competence (Allen et al., 2008). On the other hand, the lack of mentalizing skills can lead to difficulties in social adaptation, rigidity of thinking, inability to exit or enter a different role model, insensitivity and problems in relationships. The lack of or poor mentalization makes overcoming the challenges during the various normative crises difficult. Mentalization can be part of the etiopathogenesis of mental disorders and be relevant to the prognosis of the outcome. Accordingly, developing mentalizing skills is essential for psychosocial well-being, forming psychotherapeutic competence and the overall process (Fonagy and Luyten, 2009). Mentalization is related to developing different scenarios regarding assumptions about significant people in relationships. This characteristic can underlie dysfunctional family dynamics. Improving the process of mentalization in the family is associated with reducing levels of aggression and violence (Asen and Fonagy, 2016, 2017).

A necessary condition for the optimal development of the mentalization process is related to low arousal levels (Varcheva, mentalization process when working with families, 2020). The presence of severe anxiety, fixation on depressive thoughts, and other intense emotional experiences, make mentalization difficult. Therefore, the individual switches to functioning which is conditioned by evolutionarily older automated patterns of behavior. The stronger the stress and emotional state are, the more likely implicit mentalizing will dominate. However, if the experience gives way to the rational nature and the individual can reflect on the current situation, he can have more balanced and flexible experience (explicit mentalization), both for his mental processes and for the emotions, thoughts, and behavior of others (Asen and Fonagy, 2017).

### The different dimensions of mentalization are

- Ability for self-reflection and self-understanding - the ability to recognize, understand and analyze one’s emotions, thoughts and reasons for a particular behavior or reaction.
- Empathy – the ability to look through someone else’s perspective, put yourself in the place of the other person, and realize the reasons for his thoughts, emotions and behavior without losing yourself. The ability to “step into somebody shoes,” to look through his eyes at his own life.
- Understanding mental complexity – human emotions and thoughts are complex and often contradictory.
- Adaptability – the capacity to be flexible and adapt in various social situations. Along with the development of skills to understand, assess, predict and interpret the reactions of others.
- Conflict regulation – the relationship between mentalizing and conflict resolution is directly proportional, as an individual with a higher level of mentalizing is more likely to seek for understanding rather than reacting emotionally.
- Helps to build up and strengthen the attachment – mentalizing plays an essential role in the process of forming a secure attachment\bond in childhood, which subsequently serves as a foundation for building healthy relationships later in life (Slade, 2005).

Mentalization is critical for various aspects of human behavior: interpersonal relationships, communication, parenting, and professional life. Lack of ability to mentalize or problems in this process can lead to difficulties in interpersonal communication, overcoming accumulated stress and social adaptation. Such problems are also found in some mental disorders: pathological personalities, eating disorders, stress-related disorders, etc. In psychotherapy, many approaches have been developed which make a focus on improving the mentalizing skills of the patients. Developing this ability is critical for our psychosocial well-being. It allows us to connect deeply emotionally with understanding the complex world of human emotions and thoughts (Bateman and Fonagy, 2016).

## Biological, psychological, and social aspects of mentalization

Mentalization is a complex process that is made up of many factors which can be classified into three main categories:

### Biological aspects

- Brain structures thought to be involved in the process of mentalization are insula, medial prefrontal cortex and temporoparietal lobe. A lot of different methods are used to reveal the mechanisms of those processes, for example functional magnetic resonance imaging that shows particular neuronal activity which has a key role in the process of mentalization (McAdams et al., 2018).
- Hypothesized that the neurotransmitters in this process are oxytocin, serotonin and dopamine (Slade, 2005).
- It is assumed that the different penetrance of multiple genes which has different levels of expression are responsible for mentalization to be a continuum, and everyone has its place.

### Psychological aspects

- The level of parental mentalization is related to forming secure attachments in early relationships and the child’s better skills in forming meaningful contacts in the future (Berthelot et al., 2019).
- The successful path for children who are going through normative crises, and subsequently through various situational and personal dilemmas, is also associated with a better level of developed mentalization (Fonagy et al., 2002). And so forth, the accumulated psychological and time-resistant experiences would reflect harmonious to the development, together with not bearing the marks of deficiency (Fonagy and Target, 1997).

### Social aspects

- Different cultures can reveal different meaning to the ability of mentalizing and conditioning or block its development (Luyten et al., 2017). There is simple evidence that childhood emotional abuse can reach up to 48% of the general population in various forms of aggression (Goldsmith et al., 2013). This childhood trauma is associated with difficulty in mentalizing later in life, borderline personality disorder, anxiety and depression disorder (Bounoua et al., 2015). The precise mechanism by which childhood emotional trauma is associated with an increased risk of psychopathology later in life is not yet understood\revealed. However, several authors believe that mentalizing deficits represent a transdiagnostic risk for psychopathology (Fonagy et al., 2016).

## Relationship between mentalization and social realization

The ability to understand and interpret one’s mental world, in its complexity, has a direct relationship with the social realization of the individual. This process is directly related to more successful dealing with the challenges of specific situation and can improve personal and professional relationships. Different situations at work and school can determine the communication style with classmates and colleagues later. The better understanding and control of one’s emotions and behavior, implies the consolidated and adaptive use of the individual’s current abilities in different context, along with his more successful adaptation – psychological flexibility (Bateman and Fonagy, 2016). Mentalization is an essential aspect of an individual’s personality. Moreover, it is associated with more successful coping mechanisms with biopsychosocial challenges during adolescence and later life. This is associated with better emotional regulation, socioemotional losing his identity and his overall authentic image (in terms of experiences, intimate thoughts, behavior, etc.), connect with the significant other, empathize, be the creator of his life and realize his creative potential in various aspects of social functioning.

## Relationship between mentalization, etiology and pathogenesis of mental disorders

A good understanding and in-depth knowledge of mentalization theory plays an essential role in the context of the biopsychosocial model, as well as the understanding of some mental disorders. It is thought that differences in the ability to mentalize correlate with higher or lower incidence of certain mental disorders in different populations and groups (Sharp et al., 2016). Insufficiently developed mentalization in early childhood can be related to experienced trauma, abuse or neglect of the child. Those early experiences have been linked to the development certain mental disorders later in life. From the development of different dysfunctional attachment styles, deficits in mentalization and psychosocial predisposition to certain mental disorders (for example, borderline personality disorder). Some researchers indicate that neurobiological factors such as structural and functional abnormalities of the brain are associated with mentalizing problems. Deficits in this process can lead to difficulties in social relationships, social isolation, and social conflict, contributing to developing resistance to mental disorders (Choi-Kain and Gunderson, 2008). Moreover, there are progressively more studies which emphasize the relation between diagnoses like schizophrenia, personality disorders, eating disorders, and their prognoses and outcome by means of building mentalization skills (Dimopoulou et al., 2017; Volkert et al., 2019; Zettl et al., 2020; Jewell et al., 2023).

## Relationship between mentalization and the prognosis of mental disorders

Patients’ ability to mentalize can facilitate the communication with the psychiatrist and psychotherapist, improve adherence and compliance and make therapy more effective. Understanding one’s emotions and thoughts is associated with a better prognosis and outcome of the treatment. Mentalization improves interpersonal contacts, which have positive effects. It is related to the extension and assistance of the social support system, the processing of stigma and readaptation, as well as resocialization. Absent or reduced mentalizing ability is associated with more frequent relapses and recurrence in some mental disorders (Choi-Kain and Gunderson, 2008).

## Mentalization and the ability to process the stigma of mental disorder

Mentalization is related to stigma processing in two main ways: it helps others to understand what people with mental disorders are going through, which also improves tolerance and empathy. Reduce generalization and labeling. On the other hand, it helps to patients to understand the cause of their symptomatic and mental distress, to develop more adaptive coping mechanisms, or to implement a better transition to ‘living with the illness’. By understanding the inner world of the other, prejudices and stereotypes are mitigated and can even be destroyed, along with not actively participate in stigmatization (Corrigan, 2004). Increasing emotional support from relatives and society to patients with mental disorders has a crucial role in the process of healing. Applying mentalization in educational and therapeutic modalities can increase awareness and understanding of the nature of mental disorders. Furthermore, it can reveal different ways to help patients (Livingston and Boyd, 2010).

## Mentalization and psychotherapeutic competence

In psychotherapy, mentalization takes a fundamental role in building up connections, understanding, and interpreting the thoughts, feelings, and behaviors of patients/clients, along with achieving successful therapeutic outcomes. Therapists at high levels of mentalization are most likely to establish meaningful and trusting psychotherapeutic contact. This is important for building a deep and satisfying therapeutic relationship, as well as it is often associated with better outcome of psychotherapy treatment (Fonagy et al., 2002).

Mentalization is associated with several psychotherapeutic skills:

- Empathy – understanding with distancing. Mentalization and empathy are two critical psychological concepts in building a therapeutic alliance. The ability to “see” and understand the inner psychic world of the client (and other people), understanding the psychic pain in the context of the conflict dynamics of the client/patient, and the available potential for development and change. Empathy and mentalization are complementary (Decety and Jackson, 2004). While mentalization focuses on the intellectual understanding of another’s thoughts and emotions (rational nature and secondary capabilities), empathy focuses on the emotional perception of others’ experiences (emotional nature and primary capabilities). In psychotherapeutic competence, mentalization helps in understanding patient/client, while empathy allows the therapist to establish an authentic, congruent and deep emotional connection.
- Reflectiveness – this is the ability of the therapist to reflect on his own reactions and emotions about the patient. Mentalization and the capacity for self-reflection are essential psychological concepts in psychotherapy and self-awareness. Although both relate to understanding the inner psychic world of the other, they have different emphases. In mentalizing, understanding unfolds through one’s own cognitive and emotional resources. Self-awareness (self-reflection) relates to the ability of the therapist and the client/patient to consider, analyze and evaluate their feelings, thoughts and behavior. It is a process of self-observation and self-evaluation – a process focused exclusively on the self (Morin, 2006). Mentalization and self-reflection are developing in early childhood and are continuing to be enriched throughout life. This characteristic of theirs also represents the hidden psychotherapeutic potential and can be related to the brain’s neuroplasticity. When the child is born, “the parent is born” with his behavior and the ability to mentalize and influence the upbringing and formation of these abilities in the child. In psychotherapy, the therapist discovers, forms an develops these capacities (empathy, mentalization and self-reflection) in patients/clients. For this reason, there is no psychotherapy without a client. Psychotherapy, once started, is forever; only meetings with the therapist have a beginning and an end.
- Transference and countertransference – this is the competence to understand how personal stories and emotions influence the therapeutic process (Racker, 2007). These constructs are related with each other and are essential in understanding the psychotherapeutic setting. Mentalizing helps the therapist to understand the transference reactions of the patient/client and interpret the feelings and thoughts behind them (Racker, 2007). Mentalization is the basis of the therapist’s ability to self-reflect, recognize, and process his countertransference reactions. In this way, he increases his professional efficiency with the client/patient and in the supervision process. Thus, the psychotherapeutic relationship is strengthened, and the therapist becomes more sensitive to the needs of clients/patients (Gabbard, 2014).

## Mentalization and psychotherapy outcomes

In the context of different psychotherapy modalities, the psychotherapist’s ability to mentalize is associated with better therapeutic outcomes, which are related to:

- Development of the ability to better understand oneself in terms of one’s thoughts, emotions, and needs. To recognize them, to name them, to satisfy them, and to build sufficient boundaries to uphold them. This process helps in resolving the internal conflict dynamics of clients/patients (Bateman and Fonagy, 2016).
- Developing mentalization helps communication to be more effective. Initially, this process takes place between the client /patient and the therapist, and subsequently, it is brought out of the therapeutic process into the social reality of the individual. In this way, one can address one’s thoughts and feelings toward others more precisely and comprehensibly.
- Experiencing various psych traumas/ psychological traumas during an individual’s life leads to difficulties in mentalization, which further impairs the individual’s ability to overcome what has happened and can lead to a defragmentation of the self. For this reason, restoring the client’s/patient’s ability to mentalize significantly improves the crisis’s resolution and elicits a positive psychological experience (Racker, 2007).
- The ability to mentalize can help to more effectively recognize and process transference and countertransference reactions in psychotherapy (Allen et al., 2008).
- Mentalization helps to a great extent to understand one’s motivation and the reasons for their behavior, which is critical to initiating changes in the client/patient. On the other hand, taking responsibility for one’s own life supports a higher level of motivation to adhere to new ways of behaving (Morin, 2006).

## Conclusion

From the information presented so far, we could conclude that the construction of mentalization in the individual, family system and society could bring about several positive changes:

- Improved ability to recognize and name one’s thoughts, emotions and needs. Verbalizing them to the significant other and setting healthy boundaries.
- Improves the ability to understand and validate the thoughts, feelings and needs of others. Ability to satisfy them, respect, and respect foreign borders.
- Ability to relate to the other’s experience, differentiation, and separation without generating fear of separation.
- Realizing, accepting, and taking responsibility for ourselves and others we enter into relationships with.
- A better and holistic understanding of clients/patients’ symptomatic and psychological distress.
- Ability to conceptualize and predict prognosis and obstructions in the diagnostic and therapeutic process.
- Building psychotherapeutic competence and empathic conduct of psychotherapy in the primary psychotherapeutic interview and process. Successful outcome from psychotherapy and post- therapy growth.

## Author contributions

ZA: Conceptualization, Supervision, Validation, Visualization, Writing – original draft. RP: Data curation, Investigation, Resources, Validation, Writing – review & editing.
